# Laparoscopic sacrocolpopexy versus vaginal sacrospinous fixation for vaginal vault prolapse, a randomized controlled trial: SALTO-2 trial, study protocol

**DOI:** 10.1186/s12905-017-0402-2

**Published:** 2017-07-26

**Authors:** Anne-Lotte W. M. Coolen, Mèlanie N. van IJsselmuiden, Anique M. J. van Oudheusden, J. Veen, Hugo W. F. van Eijndhoven, Ben Willem J. Mol, Jan Paul Roovers, Marlies Y. Bongers

**Affiliations:** 1 Department of Obstetrics and Gynaecology, Máxima Medical Centre Veldhoven, De Run, 4600 5500 MB Veldhoven, The Netherlands; 2 Department of Gynaecology and Obstetrics, Isala Zwolle, Dokter van Heesweg 2, 8025 AB, Zwolle, The Netherlands; 3 Department of Gynaecology and Obstetrics, The Robinson Research Institute | School of Paediatrics and Reproductive Health, University of Adelaide, 5000 SA Adelaide, Australia; 4 Department of Gynaecology and Obstetrics, Academic Medical Centre Amsterdam, Meibergdreef 9, 1105 AZ Amsterdam, the Netherlands; 5 Department of Obstetrics and Gynaecology, University of Maastricht, Grow School of Oncology and Developmental Biology, Minderbroedersberg 4-6, 6211 LK Maastricht, The Netherlands

**Keywords:** Laparoscopic sacrocolpopexy, Laparoscopic sacral colpopexy, Pelvic organ prolapse, Vaginal sacrospinous ligament fixation, Vaginal sacrospinous suspension, Vault prolapse

## Abstract

**Background:**

Hysterectomy is one of the most performed surgical procedures during lifetime. Almost 10 % of women who have had a hysterectomy because of prolapse symptoms, will visit a gynaecologist for a surgical correction of a vaginal vault prolapse thereafter. Vaginal vault prolapse can be corrected by many different surgical procedures. A Cochrane review comparing abdominal sacrocolpopexy to vaginal sacrospinous fixation considered the open abdominal procedure as the treatment of first choice for prolapse of the vaginal vault, although operation time and hospital stay is longer. Literature also shows that hospital stay and blood loss are less after a laparoscopic sacrocolpopexy compared to the abdominal technique.

To date, it is unclear which of these techniques leads to the best operative result and the highest patient satisfaction. Prospective trials comparing vaginal sacrospinous fixation and laparoscopic sacrocolpopexy are lacking. The aim of this randomized trial is to compare the disease specific quality of life of the vaginal sacrospinous fixation and laparoscopic sacrocolpopexy as the treatment of vaginal vault prolapse.

**Methods:**

We will perform a multicentre prospective randomized controlled trial. Women with a post-hysterectomy symptomatic, POP-Q stage ≥2, vaginal vault prolapse will be included. Participants will be randomized to the vaginal sacrospinous fixation group or the laparoscopic sacrocolpopexy group.

Primary outcome is disease specific quality of life at 12 months follow-up. Secondary outcome will be the effect of the surgical treatment on prolapse related symptoms, sexual functioning, procedure related morbidity, hospital stay, post-operative recovery, anatomical results using the POP-Q classification after one and 5 years follow-up, type and number of re-interventions, costs and cost-effectiveness. Analysis will be performed according to the intention to treat principle and not as a per protocol analysis. With a power of 90% and a level of 0.05, the calculated sample size necessary is 96 patients. Taking into account 10% attrition, a number of 106 patients (53 in each arm) will be included.

**Discussion:**

The SALTO-2 trial is a randomized controlled multicentre trial to evaluate whether the laparoscopic sacrocolpopexy or vaginal sacrospinous fixation is the first-choice surgical treatment in patients with a stage ≥2 vault prolapse.

**Trial registration:**

Netherlands Trial Register (NTR): NTR3977; Registered 28 April 2013.

## Background

Hysterectomy is one of the most performed surgical procedures during a women’s lifetime. Almost 10% of women who have had a hysterectomy because of prolapse symptoms, will visit a gynaecologist for a surgical correction of a vaginal vault prolapse (VVP) thereafter. VVP can be corrected by many different surgical procedures. These symptoms are directly related to the prolapse and contain of pelvic pressure, bulging of the vaginal wall, dropping sensation in the vagina or backache. Other symptoms that are often present, are symptoms of the bladder, bowel and sexual problems [1]. These symptoms could affect the quality of life of these women severely. Therefore, an effective treatment is required.

The incidence of post-hysterectomy VVP requiring surgical treatment, has been estimated at 36 per 10,000 person-years [2]. The longer the time after hysterectomy, the higher the risk of vault prolapse. If the initial reason for hysterectomy was genital prolapse the risk increases significantly [1–3]. Women tend to get older and older and due to this improved life expectancy, there will be an enormous extra demand for future prolapse surgery.

Surgery for pelvic organ prolapse, including VVP, focuses on the correction of the normal anatomy of the vagina, resulting in normal function of the bladder and bowel. To date, a variety of surgical interventions to treat VVP surgically have been described [4]. These procedures can be performed vaginally or abdominally. The abdominal route can be performed as an open or laparoscopic sacrocolpopexy (LSC). The vaginal approach includes the vaginal sacrospinous fixation (VSF), which was first reported in 1958 [5]. This is probably the most performed treatment modality of VVP at the moment. In a questionnaire of the International Urogynecological Association (IUGA), which was performed in 2002, VSF was the most performed surgical correction for the VVP, as 78% of the responders reported the VSF as the first-choice treatment for VVP [6]. The LSC technique was developed in the footsteps of the abdominal sacrocolpopexy, and has, been implemented since then [7].

No randomized controlled trials comparing VSF and LSC have been performed. A Cochrane review shows that abdominal sacrocolpopexy is better compared to VSF. The recurrence rate of VVP was lower after an abdominal sacrocolpopexy (RR 0.23, 95% CI 0.07 to 0.77) and dyspareunia was less (RR) 0.39, 95% CI 0.18 to 0.86). However, the rates of recurrence surgery for prolapse show no statistical difference (RR 0.46, 95% CI 0.19 to 1.11). The VSF has a shorter operation time, lower costs and an earlier return to daily activities [8]. In none of the included studies disease specific quality of life was the primary outcome. Furthermore, in some of the studies no power analysis was done.

A cohort study comparing laparoscopic to abdominal sacrocolpopexy shows a significant reduction in hospitalization (1.8 ± 1.0 days vs 4.0 ± 1.8 days; *p* < 0 .0001) [9]. A prospective cohort study comparing laparoscopic to abdominal sacrocolpopexy that we performed prior to this study revealed a significant reduction in blood loss (77 ml (±182) versus 192 ml (±126) respectively, p = <.001) and hospital stay (2.4 days versus 4.2 days respectively, p = <.001) and less procedure related morbidity (RR 0.24 (95%-CI 0.07–0.80), *p* = 0.009) [10].

The laparoscopic procedure seems to have advantages over the abdominal procedure.

Since prospective trials comparing VSF and LSC sacrocolpopexy are lacking we plan to perform a RCT. The aim of this randomized trial is to compare the disease specific quality of life of the VSF and LSC as the treatment of VVP.

## Methods/study design

### Study design

The SALTO-2 trial is a randomized controlled multicentre trial and will be performed to compare VSF versus LSC for VVP. The follow-up time will be one and 5 years.

**Fig. 1:**
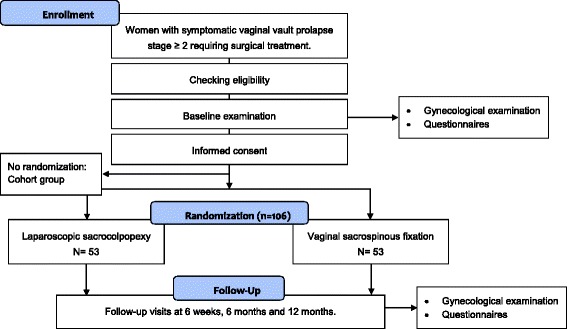
Study design

The trial will be a non-blinded trial, since it is impossible to blind the participating women and medical staff for the allocated technique, since one procedure will be performed vaginally and the other one laparoscopically, leaving small abdominal scars. However, a physician blinded for the intervention will perform follow-up examination. This will be another physician than the surgeon who performed the operation. The study design is presented in Fig. 1.

### Objectives

The objective of this study is to determine whether LSC in women with vault prolapse, POP-Q stage 2 or higher, improves outcome in terms of disease specific quality of life, recurrence of prolapse, complications, hospital stay, post-operative recovery, sexual functioning, costs and costs-effectiveness, compared to VSF.

### Hypothesis

Based on the literature, we expect that the LSC will be equally or more successful in correction of vault prolapse and its related disease specific quality of life as compared to VSF.

### Participating hospitals

The trial will be performed in several teaching and academic hospitals in the Netherlands. The nine participating centres are Máxima Medical Centre, Isala Clinics, Spaarne Gasthuis, Catharina Hospital, Maastricht Academic Hospital, Gelre Hospital, Radboud Academic Hospital, Sint Lucas Andreas Hospital, VU Medical Centre and Martini Hospital. Before the start of the trial, a masterclass was organised to reach consensus on the details of operation technique of the LSC and VSF and evaluate the operation skills of the participating surgeons. All participating gynaecologists performed at least 25 procedures before the beginning of the trial to exclude a learning curve.

During this master class, which was attended by many experienced surgeons, several surgical steps of both procedures were discussed (for the sacrocolpopexy: type of mesh, type of sutures, number of sutures, dissection technique, re-peritonealisation, (no) obliteration of Douglas pouch. For the VSF: (no) hydro dissection type and number of sutures, concomitant prolapse surgery). Decisions which techniques should be used were made and recorded to reduce practice variation as much as possible and to carry out a uniform operation technique during the inclusion period.

### Study population and recruitment

All patients with a symptomatic post-hysterectomy VVP stage 2 or higher (according to POP-Q classification) who need surgical treatment are eligible for the study.

#### Inclusion criteria

In order to be eligible to participate in this study, a subject must meet all of the following criteria:

- Symptomatic vault prolapse POP-Q ≥ stage 2 which needs surgical treatment
- Eligible for both surgical treatments
- Patients must be able to read Dutch

#### Exclusion criteria

A potential subject who meets any of the following criteria will be excluded from participation in this study:

- Previous surgical treatment of vault prolapse
- Contra-indication for a surgical intervention
- Incapacitated patients, illiterate patients or patients with other language barriers

Patients with co-existing anterior/posterior defects or concomitant incontinence surgery can be included. Patients need to agree to return the questionnaires and visit the follow-up appointments.

Patients who don’t want to participate in the trial because of a preference for one of both surgical options will be asked for a cohort group and requested to complete the questionnaires as well. This cohort group will be compared to the study population to analyse whether a patients preference will affect the quality of life.

Assessment for eligibility will be performed by a gynaecologist of the participating hospital. Women eligible for this trial will be counselled for the trial. Subsequently, written patient information is provided, which contains information on the objectives, design, methods, possible advantages and disadvantages of the study treatments, and information that non-co-operation with the study or withdrawal will not have consequences for their treatment. Before randomization, written informed consent will be obtained.

### Interventions

#### Vaginal sacrospinous fixation

The patient is placed in lithotomy position. The sacrospinous ligament will be accessed through an incision following the length of the posterior vaginal wall, extending up to the vaginal vault. Blunt dissection is used to open the right pararectal space and locate the ischial spine. A ‘window’ is created through the rectal pillar, large enough for two fingers. Just lateral to the rectum and above the puborectal muscle, the right sacrospinous ligament-coccygeus muscle complex will be exposed. Three Breisky specula will be positioned, whereafter two Prolene 1–0 sutures will be placed under direct vision. These two permanent non-absorbable sutures will be put into the sacrospinous ligament at about 0.5 cm apart, with the lateral suture being placed about 2 cm from the ischial spine. The sutures will be attached to the vault on the suture line were the vault was closed after hysterectomy seeking the part with most connective tissue or ligament remains.

#### Laparoscopic sacrocolpopexy

Patients don’t receive bowel preparation the day before the operation. Looking at the design of this surgical intervention, the main goal of sacrocolpopexy is to reconstitute an adequate, durable system of support and suspension of the vagina by replacing the impaired and/or detached native fascial tissue with a synthetic non-absorbable prosthesis. The LSC will be performed under general anaesthesia with four trocars, one for the scope and three side trocars. The vaginal vault will be lifted using a vaginal probe. The peritoneum will be dissected to expose the vesicovaginal and rectovaginal fascia, extending to the sacral promontory. Preparation of the rectovaginal and vesicovaginal fascia will be done as far down as possible. The preparated tissue and the size of the mesh will be measured and documented. One side of the polypropylene mesh will be attached anteriorly of the vaginal wall, and the other side as far down posteriorly as possible using absorbable sutures. As little as possible stitches will be used. Depending on the surgeon’s preference, the mesh will be attached to the sacral promontory using staples or non-absorbable sutures. The mesh will be peritonealised at several points. The pouch of Douglas will not be obliterated.

The VSF can be performed under spinal or general anaesthesia, depending to the patient’s and anaesthesiologist’s preferences. The laparoscopic procedure will be performed under general anaesthesia. Both procedures will be completed with any additional vaginal surgery, if indicated, after the vault suspension has been carried out. For example anterior and posterior colporrhaphy may be performed during the same procedure. No vaginal mesh augmented procedures are allowed.

In both groups, prophylactic antibiotics and thrombosis prophylaxis will be given per-operatively. An indwelling urine catheter will be left in-situ and will be removed the first day post-operatively or as clinically indicated. Prolonged catheterisation will be recorded. If necessary, patients will receive analgesics according to the local hospital protocol. Patients are advised to withhold from heavy physical work for a minimal period of 6 weeks.

In case clinically indicated (complication or technical challenge to continue the procedure), the surgeon could convert to the other intervention. Participants will be analysed according to the intention to treat principle.

### Data collection

Participants will be followed pre-operatively, until one and 5 years post procedure. At follow-up, several aspects will be evaluated:

- Clinical examination of the prolapse using POP-Q.
- UDI, the Dutch validated version of the Urinary Distress Inventory, questionnaire comprising 17 questions, to assess the presence and experienced discomfort of pelvic floor problems. The UDI consists of 5 domains: discomfort/pain, urinary incontinence, overactive bladder, genital prolapse, and obstructive micturition. The total UDI score is defined as the average of the 5 domain scores, and can be used to assess cost effectiveness by measuring quality of life (van der Vaart 2003 [11]).
- DDI, the Defecatory Distress Inventory is a standardized questionnaire measuring defecatory symptoms. The questions cover the following sections: obstructive defecation, constipation, fecal incontinence and pain related to defecation. Patients have more bothersome symptoms if they have a high score on a particular section. (Roovers 2008 [12]).
- IIQ, the Incontinence Impact Questionnaire is a disease-specific quality of life questionnaire covering the five sections: physical functioning, mobility, emotional functioning, social functioning and embarrassment (van der Vaart 2003 [11]).
- EQ-5D, EuroQol, is a general quality of life questionnaire, to evaluate health utilities and the corresponding quality adjusted life years (QALYs). This is the difference in quality of life caused by the treatment multiplied by the duration of treatment effect (Dolan 1997 [13], Lamers 2005 [14]).
- Medical cost questionnaire.
- PISQ, Pelvic Organ Prolapse/Incontinence Sexual Questionnaire, to analyse sexual function in participants with urinary incontinence and/or pelvic organ prolapse (Occhino 2011 [15]).
- PGI-I, Patient Global Impression of Improvement, to evaluate the post-operative condition as compared to the condition before the surgical intervention. A single question is used to rate the condition, and the answer can be given on a scale from “1. Very much better” to “7. Very much worse” (Srikrishna 2010 [16]).
- Pre-operative urodynamic examination is only necessary when clinically indicated.
- During the first 6 weeks post-operative (including the hospitalization), participants
- are asked to keep a diary, which includes the following sections: postoperative pain measured by Visual Analogue Score (VAS), used pain medication and the RI-10 recovery questionnaire. RI-10, the Recovery Index 10 is a questionnaire evaluating post-operative recovery. The questionnaire consists of 10 items using a 5 point-Likert scales. (Kluivers 2008 [17]).
- To evaluate post-operative recovery and satisfaction three questions are added to the 12-month questionnaire:
  1. Are you satisfied with the post-operative result?
    Answers: yes/no/don’t know
  2. Did the operation improve your symptoms?
    Answers: yes/no/don’t know.
    Would you recommend the surgery to a friend?
    Answers: yes/no/don’t know.

*After inclusion, the following data will be recorded:*

**Table:** 

	POP-Q	UDI	DDI	IIQ	EQ-5D	Medical costs	PISQ	PGI-I	Diary	Satisfaction questionnaire
Baseline	x	x	x	x	x	x	x	-	-	-
6 weeks	x	-	-	-	-	x	-	-	x	-
6 months	-	x	x	x	x	x	x	-	-	-
1 year	x	x	x	x	x	x	x	x	-	x
5 years	x	x	x	x	x	x	x	x	-	x

Randomized participants will be scheduled for follow-up visits pre-operatively, at 6 weeks and one and 5 years post-operative. During these out-patient visits a physical examination including POP-Q will be performed and complications will be detected. The follow up visit at one and 5 years will be performed by a physician blinded for the intervention. This will not be the surgeon who performed the operation.

Post-operative recovery will be assessed by asking the patients to keep a diary during their hospital stay and in the first 6 weeks post-operative. The diary consists of the several sections: VAS pain score, pain medication and the RI-10 recovery questionnaire. A part of the questionnaire of the economic evaluation is also added to the diary.

Secondary outcome will be the effect of the surgical treatment on prolapse related symptoms, post-operative recovery, procedure related morbidity, sexual function, quality of life, anatomical results using the POP-Q classification until 1 year follow-up, type and number of re-interventions, costs and cost-effectiveness and long term complications. Other study parameters are:

- procedure time
- blood loss
- hospital stay
- post-operative pain medication
- post-operative pain score (visual analogue scale)
- peri-operative complications

Other study parameters are baseline values or parameters which might intervene with the main study parameter, like duration of symptoms, medical history, parity, body mass index, education/profession, smoking, atrophy, pre- or postmenopausal status, use of oestrogens or hormone replacement therapy, previous prolapse or stress incontinence surgery, previous pessary therapy, combined prolapse- or stress incontinence surgery and type of sutures and mesh during the intervention.

In case of loss to follow-up, participants will be contacted by telephone and asked for the reason for not returning the questionnaires or returning for follow-up visits. If necessary, the general practitioner will be contacted to gather additional information. Characteristics of responders and non-responders will be compared.

### Economic evaluation

The costs of both surgical treatments will be compared. The direct costs of the VSF and LSC, like costs of operating time and use of materials, will be taken into account. Moreover, medication for post-operative pain reduction, length of hospital stay and admission for complications or re-interventions will be assessed. The economic evaluation will be conducted from a societal perspective including direct medical and direct non-medical costs. Home care, consisting of both professional care as well as informal or family care will be evaluated. We will use a patient questionnaire to collect all the information of the additional home care. This questionnaire is added to the diary which will be kept by all patients. Productivity losses will not be included in the economic evaluation, since most of the participants will be over 55 years of age. To gather medical costs a case record form will be used. Cost components will be valued according to standard Dutch guidelines for economic evaluation (CVZ 2004). Actual costs will be estimated for the VSF and LSC and informal care will be valued by using shadow prices. These data will be used to perform a cost-effectiveness analysis.

To perform a cost-utility analysis, we will use the EuroQol questionnaire (EQ-5D). This is a disease non-specific quality of life questionnaire, to derive health utilities and the corresponding quality adjusted life years (QALYs). This is the change in quality of life induced by the treatment multiplied by the duration of treatment effect. QALYs can then be related to medical costs to arrive at a final common denominator of cost/QALY.

### Primary and secondary outcomes

The primary outcome is the functional effect by evaluating disease specific quality of life at 12 months follow-up using the Dutch validated version of the Urinary Distress Inventory (UDI). Secondary outcome will be the effect of the surgical treatment on other prolapse related symptoms as defecation and sexual problems and the anatomical results using the Pelvic Organ Prolapse Quantification (POP-Q) at one and 5 years follow-up. Other secondary outcomes are procedure related parameters as procedure time, estimated amount of blood loss, length of hospitalization, post-operative pain medication, post-operative pain score (visual analogue scale) and peri-operative complications, post-operative recovery, general quality of life, type and number of re-interventions, costs and cost-effectiveness and long term complications. Another secondary outcome will be the success rate according to Barbers’ criteria. Success is defined as no prolapse of the vault beyond the hymen, no bothersome bulge symptoms (vaginal bulging and protrusion according to the validated questionnaire), and no repeat surgery or pessary use for recurrent vault prolapse [18].

### Sample size calculation

We will consider the score of the UDI genital prolapse domain as primary endpoint. A difference between both surgical techniques of 10 points on the genital prolapse domain of the UDI 1 year after surgery, will be considered a clinically relevant difference between both groups [19]. The standard deviation of the score on this domain is 15 points [19]. With a power of 90% and a level of 0.05, the calculated sample size necessary is 96 (48 in each group).

The analysis will be performed by intention to treat. Odds ratios and 95% confidence intervals are calculated for all terms that are included in the regression model. Domain scores will be analysed using repeated measurement analysis.

Taking into account 10% attrition, a number of 106 patients (53 in each arm) will be included.

### Randomization

The trial represents a multi-centre randomized controlled design. Eligible patients with vault prolapse who meet the inclusion criteria will be randomized when informed consent is signed. The treatment allocation ratio is 1:1 to either LSC or VSF. Stratified randomization will be used to achieve approximate balance of participating centres across study groups. The investigators or the participating surgeons are not aware of these series. Randomization will be performed by the coordinating researcher, after which the procedure can be planned. For randomization, opaque sealed envelopes will be used in order to conceal the allocation. To evaluate data anonymously, participants will receive a case number at randomization. Blinding for allocation of treatment is impossible because of the laparoscopic or vaginal approach which requires a different introduction and anaesthesia technique. However, the follow up visit at one and 5 years will be performed by a physician blinded for the intervention.

### Statistical analysis

#### Data analysis

Data will be analysed based on intention to treat principle and stratified for centre. If the treatment effect is homogenous across centres we will also perform an un-stratified analysis. To examine differences between groups we use an unpaired T-test for continuous variables and a Chi-square or, if opportune, a Fisher’s exact test for dichotomous variables.

For differences in UDI, DDI and IIQ domain scores, a repeated measurement analysis will be performed. Repeated measurements analysis provides information of the results over time. Two-sided significance tests will be used throughout. A *P* value of <0.05 will be considered to be statistically significant. Time to re-intervention will be compared with Cox regression and Kaplan Meier analysis. The statistical package used was SPSS 22.

### Ethics

The study will be carried out in accordance with the principles of the Declaration of Helsinki. The SALTO-2 trial was approved in March 2014 (version 3.1) by the Ethics Committee of the Máxima Medical Centre Veldhoven (METC 1324) and the local board of directors of the participating centres Isala Clinics, Spaarne Gasthuis, Catharina Hospital, Maastricht Academic Hospital, Gelre Hospital, Radboud Academic Hospital, Sint Lucas Andreas Hospital, VU Medical Centre and Martini Hospital. Informed consent will be obtained before participants will be randomized. Participants are currently being recruited and enrolled. The date of first enrolment was 27.09.2013. If any important modifications will be made to the protocol, an amendment will be presented to the Medical Ethics Committee of the Máxima Medical Centre Veldhoven for consideration.

## Discussion

LSC and VSF are generally performed procedures in pelvic care clinics all over the world. Although there is some literature about both surgical procedures, there is much heterogeneity in study populations and interventions. Furthermore, quality of life, which is the most relevant outcome to evaluate the effect of prolapse surgery, was no primary outcome of any of these studies [5, 6, 10]. In our opinion the question which surgical intervention leads to the highest patient satisfaction for women with a stage 2 or higher VVP is still unanswered. Prospective trials comparing disease specific quality of life after VSF and LSC are lacking. Therefore, a sufficiently powered randomized controlled trial with long-term follow-up is required to provide evidence based decisions on the preferred treatment.

## Abbreviations

DDI: Defecatory Distress Inventory, disease-specific quality of life questionnaire
EQ-5D: EuroQol, quality of life questionnaire
EU: European Union
IC: Informed Consent
IIQ: Incontinence Impact Questionnaire, disease-specific quality of life questionnaire
IUGA: International Urogynecological Association
LSC: Laparoscopic sacrocolpopexy
METC: Medical research ethics committee (MREC); in Dutch: medisch ethische toetsing commissie (METC)
NFU: Dutch Federation of University Medical Centres; in Dutch: Nederlandse Federatie van Universitair Medische Centra
NVOG: Dutch Society of Obstetrics and Gynaecology; in Dutch: Nederlandse vereniging voor obstetrie en gynaecologie.
OSAT: On-Site Assessment and Training
PISQ: Pelvic Organ Prolapse/Incontinence Sexual Questionnaire
POP-Q: Pelvic Organ Prolapse Quantification
RI-10: Recovery Index 10, post-operative recovery questionnaire
Sponsor: The sponsor is the party that commissions the organisation or performance of the research, for example a pharmaceutical company, academic hospital, scientific organisation or investigator. A party that provides funding for a study but does not commission it is not regarded as the sponsor, but referred to as a subsidising party.
SPSS: Statistical Package for the Social Sciences
UDI: Urinary Distress Inventory, disease-specific quality of life questionnaire
VAS: Visual Analogue pain Score
VSF: Vaginal sacrospinous fixation
WMO: Medical Research Involving Human Subjects Act; in Dutch: Wet Medisch-wetenschappelijk Onderzoek met Mensen
